# Diagnostic Detour: A Case of Opsoclonus-Myoclonus-Ataxia Syndrome Initially Misdiagnosed As Tricyclic Antidepressant Toxicity in a Child

**DOI:** 10.7759/cureus.101140

**Published:** 2026-01-09

**Authors:** Yousef M Al-Shammari, Shaikhah M Al-Shammari, Adnan Y BuAbbas, Sarah S Al-Qahtani, Omnia A Mahsoub, Mohamed E Amer, Talal A Al-Saleem

**Affiliations:** 1 Ophthalmology, Al-Bahar Eye Center, Ministry of Health, Kuwait City, KWT; 2 Pediatrics, Jaber Al-Ahmad Hospital, Ministry of Health, Kuwait City, KWT; 3 Pediatrics, Farwaniyah Hospital, Ministry of Health, Kuwait City, KWT; 4 Internal Medicine, Jaber Al-Ahmad Hospital, Ministry of Health, Kuwait City, KWT; 5 Pediatrics, Faculty of Medicine, Ain Shams University, Cairo, EGY; 6 Pediatrics, Faculty of Medicine, Zagazig University, Zagazig, EGY

**Keywords:** neuroblastoma, omas, oms score, opsoclonus-myoclonus-ataxia syndrome, paraneoplastic syndromes, tricyclic antidepressant

## Abstract

Opsoclonus-Myoclonus-Ataxia Syndrome (OMAS) is a rare pediatric neuroinflammatory disorder. Its recognition is challenging, as early manifestations may resemble toxic, infectious, or metabolic processes. Misdiagnosis and diagnostic delay remain important contributors to poor outcomes. The aim of this report is to describe an unusual case of OMAS initially mistaken for tricyclic antidepressant (TCA) ingestion and to underscore the diagnostic lessons relevant to pediatric emergency, neurology, and toxicology practice. We present a previously healthy four-year-11-month-old boy who was brought to the emergency department following the discovery of missing TCA tablets and three days of vomiting and fever. Initial findings included dehydration, confusion, urinary retention, and dilated pupils, suggesting TCA toxicity. He was treated empirically for possible meningitis while a toxicological evaluation was pursued. Over the next 48 hours, opsoclonus, hyperreflexia with myoclonus, and a wide-based gait emerged, redirecting the differential diagnosis toward OMAS. Neuroimaging and malignancy screening were unremarkable. Cerebrospinal fluid analysis showed lymphocytic pleocytosis, elevated protein (1189 mg/L), positive oligoclonal bands, reduced glucose (2.0 mmol/L), elevated lactate (2.8 mmol/L), increased lactate dehydrogenase (291 IU/L), and negative neuronal antibody testing. Immunotherapy with intravenous immunoglobulin and corticosteroids produced rapid improvement, with complete recovery by six months of follow-up. This case highlights the risk of anchoring bias in acute pediatrics, the necessity of malignancy surveillance, and the favorable impact of timely immunotherapy in OMAS.

## Introduction

Opsoclonus-Myoclonus-Ataxia Syndrome (OMAS) is a rare pediatric neuroinflammatory disorder, classically regarded as autoimmune or paraneoplastic in origin. Globally, its incidence is estimated at less than one per 1000000 children per year, with a peak age of onset between 18 and 36 months [1]. Approximately half of childhood cases are associated with neuroblastoma, making OMAS one of the most distinctive pediatric paraneoplastic syndromes [2]. The clinical phenotype is characterized by chaotic, multidirectional saccades (opsoclonus), myoclonic jerks, and a wide-based ataxic gait, frequently accompanied by behavioral dysregulation, irritability, and sleep disturbance [3,4]. Despite its distinctive triad, diagnosis remains clinical and is often delayed or confounded because presenting features overlap with infectious encephalitis, autoimmune encephalitis, metabolic disorders, or toxic ingestions [4].

Early recognition of OMAS is frequently hindered by its gradual and protean evolution. Initial symptoms, such as irritability, vomiting, confusion, or pupillary changes, can mimic more common pediatric emergencies, including toxic ingestions, infectious encephalitis, or metabolic crises [3]. In acute care settings, clinicians are particularly vulnerable to anchoring bias, in which a salient history, such as reported drug ingestion, overshadows emerging contradictory signs [5]. This cognitive pitfall may delay recognizing opsoclonus and evolving ataxia, which are pathognomonic for OMAS. 

The present case is noteworthy because a child with OMAS was initially misdiagnosed with tricyclic antidepressant (TCA) toxicity, underscoring how overlapping early features can misdirect the diagnostic process. We aim to highlight the need for flexibility in pediatric neurology, emergency medicine, and toxicology practice.

## Case presentation

Patient information and clinical presentation

The patient is a boy aged four years 11 months, previously healthy and without chronic medication use. The family reported three missing tablets of a TCA, each 25 mg, with the household drug name variably recorded as amitriptyline or clomipramine. The exact TCA could not be definitively identified because the medication container was unavailable, and the family provided inconsistent drug names. This uncertainty did not affect clinical interpretation or management. During the three days prior to presentation, the patient had received oral amoxicillin-clavulanate and paracetamol for fever and vomiting. On arrival at the emergency department on 17 May 2025, vital signs were as follows: temperature 37.5 °C, heart rate 90 beats per minute, blood pressure 90/60 mmHg, random blood glucose 69 mg/dL, and oxygen saturation 100% on room air. He appeared confused, with a dry tongue, bilaterally dilated pupils that were sluggish but reactive, and urinary retention. He was initially treated empirically for possible meningitis and monitored for suspected TCA toxicity while diagnostic evaluation was ongoing. Over the first two days in hospital, he developed opsoclonus, an unsteady, wide-based gait, myoclonic jerks, hyperreflexia, and bilateral ankle clonus with no meningeal signs or focal cranial nerve deficits. The Mitchell-Pike OMAS Rating Scale was used to assess disease severity based on otoscopic findings and clinical symptoms, revealing a pre-treatment score of 8 [6]. The clinical picture shifted toward OMAS while toxic and infectious causes were evaluated. 

Complete blood count indices, liver and renal function parameters, electrolytes, and inflammatory markers were largely within normal limits, apart from mild neutrophilia, lymphopenia, and borderline hyponatremia. Viral polymerase chain reaction (PCR) testing for influenza A/B, respiratory syncytial virus (RSV), and severe acute respiratory syndrome coronavirus 2 (SARS-CoV-2) was negative, and the autoimmune encephalitis antibody panel in cerebrospinal fluid (CSF) showed no detectable antibodies. In contrast, CSF studies demonstrated significant abnormalities, including markedly elevated protein, increased lactate, and low glucose levels (Table 1).

**Table 1:** Laboratory investigations of the patient at presentation RBC: red blood cell; ALT: alanine transaminase; AST: aspartate transaminase; CRP: C-reactive protein; CSF: cerebrospinal fluid; LDH: lactate dehydrogenase; RSV: respiratory syncytial virus; SARS-CoV-2: severe acute respiratory syndrome coronavirus 2; IgG: Immunoglobulin G; CASPR2: contactin-associated protein-like 2; AMPAR1: α-amino-3-hydroxy-5-methyl-4-isoxazolepropionic acid receptor 1; AMPAR2: α-amino-3-hydroxy-5-methyl-4-isoxazolepropionic acid receptor 2; LGI1: leucine-rich glioma-inactivated 1; GABARB1/B2: gamma-aminobutyric acid B receptor subunits 1 and 2

Investigations	Results	Result range	Unit
Blood indices			
Hemoglobin	135	110-140	g/L
RBC count	4.88	4.0-5.1	10^12^/L
Neutrophils	60.1	20-40	%
Lymphocytes	28.8	40-80	%
Monocytes	10.4	0-10.9	%
Eosinophils	0.4	0-3	%
Basophils	0.3	0-1.5	%
Platelet counts	332	200-490	10^3^/mm^3^
Liver function test			
Serum ALT	17	10-50	IU/L
Serum AST	26	10-50	IU/L
Serum creatinine	25	21-65	umol/L
Urea	2.3	1.8-6.4	mmol/L
Serum electrolytes			
Sodium (Na)	132	136-142	mmol/L
Potassium (K)	3.9	3.5-5.1	mmol/L
Chloride (Cl)	98	98-107	mmol/L
Bicarbonate	20	21-31	mmol/L
CRP	5	0-8	mg/L
PCR in nasopharyngeal swap			
Influenza A virus RNA	Negative	-	-
Influenza B virus RNA	Negative	-	-
Respiratory syncytial virus RNA	Negative	-	-
SARS-CoV-2 RNA	Negative	-	-
Autoimmune neurological diseases-encephalitis IgG Ab-CSF			
Anti-NMDAR	Negative	-	-
Anti-CASPR2	Negative	-	-
Anti-AMPAR1	Negative	-	-
Anti-AMPAR2	Negative	-	-
Anti-LG11	Negative	-	-
Anti-GABARB1/B2	Negative	-	-
CSF profile			
LDH	291	-	IU/L
Glucose	2.0	2.2-3.9	mmol/L
Lactate	2.8	0.6-2.2	mmol/L
Protein	1189	150-450	mg/L

Diagnostic assessment

**Figure 1:**
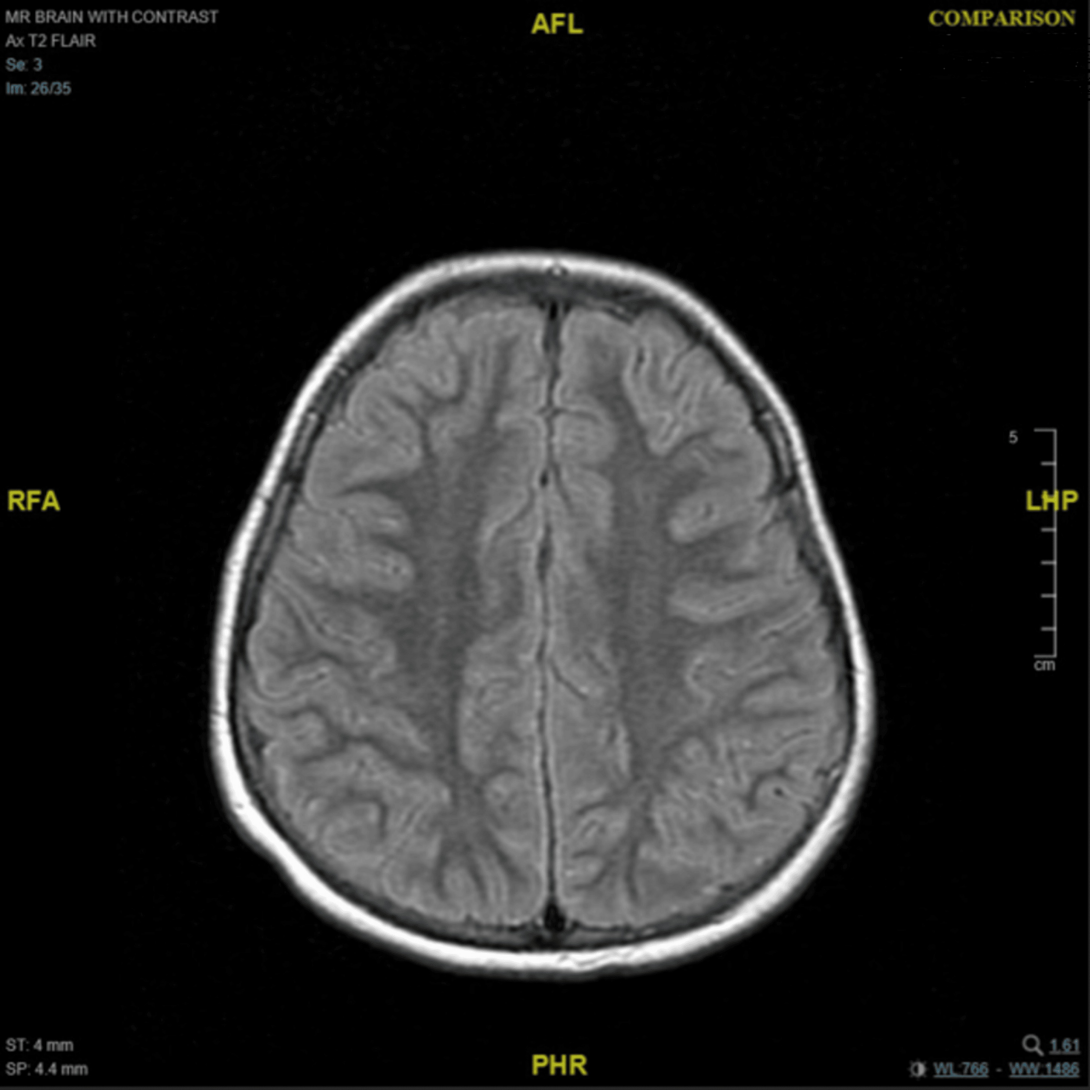
Axial T2/FLAIR brain MRI shows no definite structural or signal abnormalities FLAIR: fluid-attenuated inversion recovery; MRI: magnetic resonance imaging; T2: T2-weighted imaging; AFL: anterior-foot-left; RFA: right-foot-anterior; LHP: left-head-posterior; PHR: posterior-head-right

**Figure 2:**
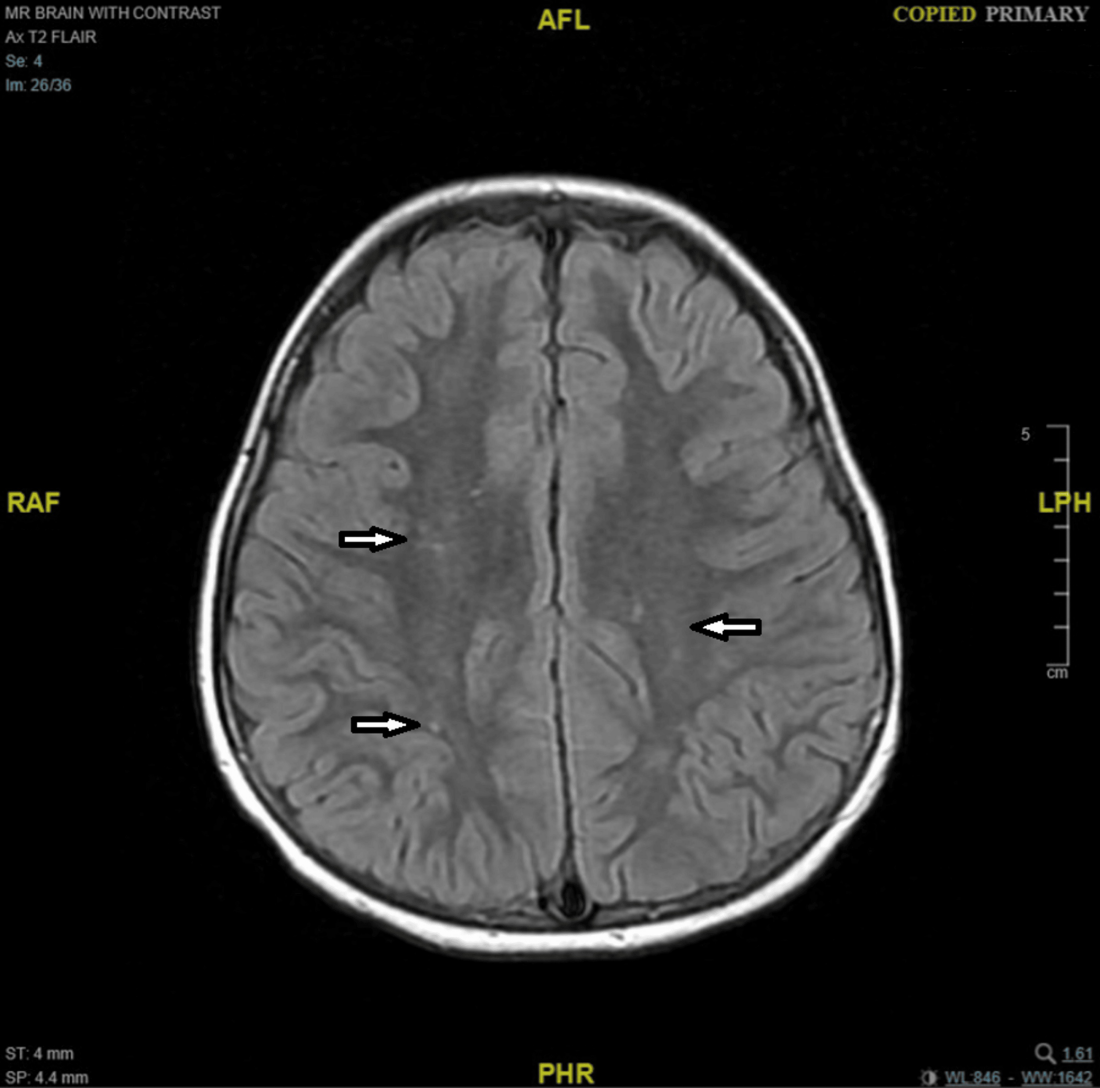
Axial T2/FLAIR brain MRI shows a few tiny scattered hyperintense foci involving the fronto-temporo-parietal subcortical white matter FLAIR: fluid-attenuated inversion recovery; MRI: magnetic resonance imaging; T2: T2-weighted imaging; AFL: anterior-foot-left; RFA: right-foot-anterior; LHP: left-head-posterior; PHR: posterior-head-right

**Figure 3:**
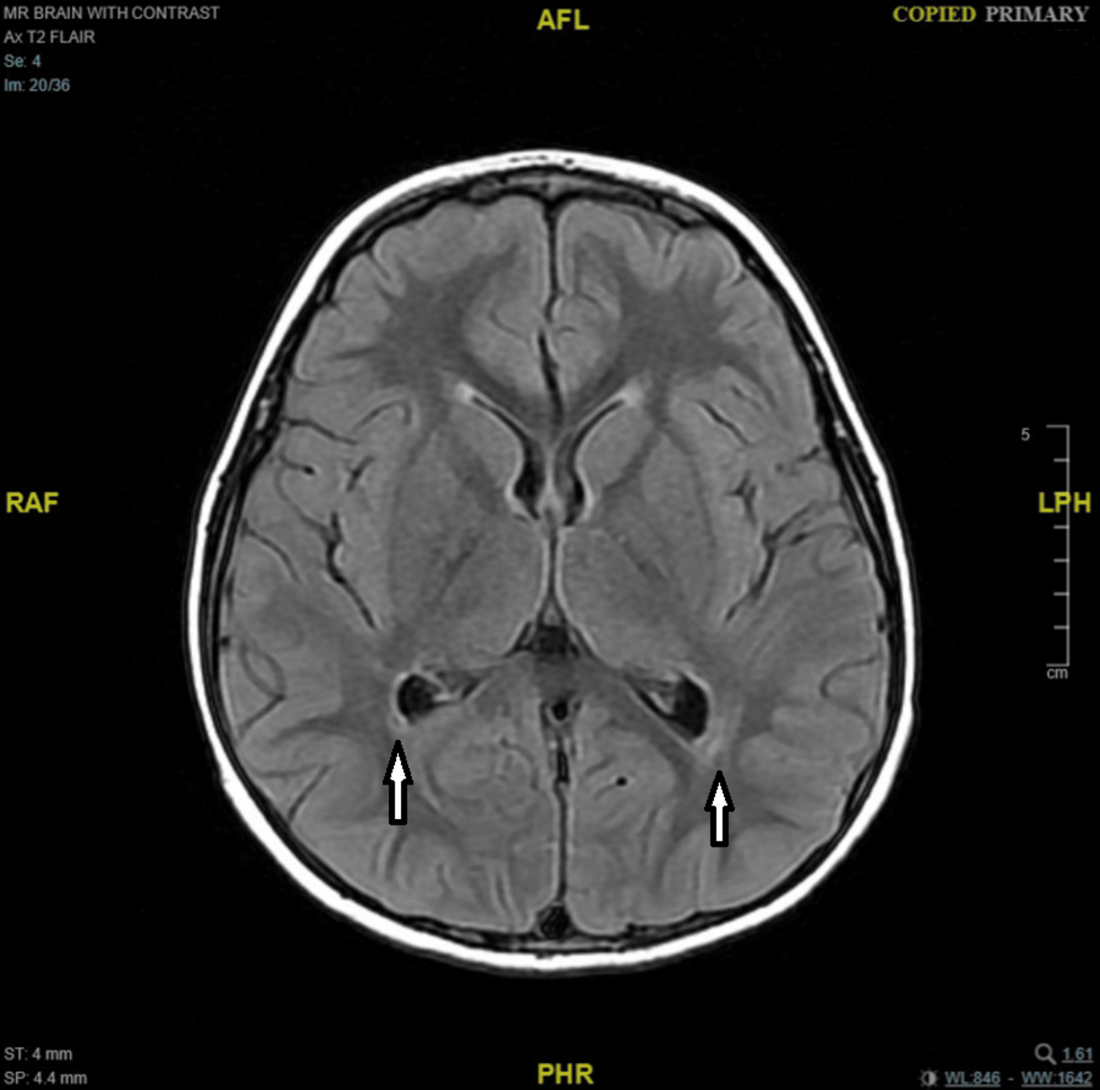
Axial T2/FLAIR brain MRI shows a hyperintense signal involving the peri-trigonal white matter FLAIR: fluid-attenuated inversion recovery; MRI: magnetic resonance imaging; T2: T2-weighted imaging; AFL: anterior-foot-left; RFA: right-foot-anterior; LHP: left-head-posterior; PHR: posterior-head-right

Neuroimaging investigations were performed sequentially. Magnetic resonance imaging (MRI) of the brain with contrast was first obtained on the third day of presentation (May 19, 2025) and was unremarkable (Figure 1). A repeat MRI of the brain and cervical spine was performed four weeks later (June 16, 2025), revealing a few adjacent terminal-zone foci of myelination with T2-weighted imaging (T2)/fluid-attenuated inversion recovery (FLAIR)/double inversion recovery (DIR) hyperintense signals in the peri-trigonal white matter, as well as a few tiny scattered T2/FLAIR/DIR hyperintense foci in the fronto-temporo-parietal subcortical white matter (Figures 2, 3). There was no diffusion restriction, contrast enhancement, or perifocal edema, and these findings were considered nonspecific or consistent with terminal zones of myelination. Tiny T2 hyperintense foci involving the infra-ganglionic regions are denoted Virchow-Robin spaces. Computed tomography (CT) of the brain was unremarkable, showing no hemorrhage, mass effect, or hydrocephalus, but a partial empty sella was noted. Abdominal and pelvic ultrasonography showed no adrenal or retroperitoneal mass. Fluorodeoxyglucose positron emission tomography (FDG-PET)/CT and fluorodopa positron emission tomography (FDOPA-PET) scans performed during the acute phase demonstrated no abnormal uptake and normal-appearing adrenal glands.

CSF analysis revealed a white blood cell count of 235 cells/mm³ (90% lymphocytes), fewer than 5 red blood cells/mm³, elevated protein at 1189 mg/L, and glucose within reference limits. The meningitis/encephalitis molecular panel and the CSF neuronal antibody panel were both negative, including NMDA, CASPR2, AMPA1, AMPA2, LGI1, and GABA antibodies. Oligoclonal bands were positive in the CSF. Serological investigations, including autoimmune antibody testing (ANA, anti-DNA), B-cell markers, tuberculosis screening, CSF culture, and urinary vanillylmandelic acid (VMA) measurement, were all within normal limits. Toxicology screening was also negative.

Therapeutic intervention

Intravenous Immunoglobulin (IVIG) (2 g/kg over five days) was initiated on day three of admission. Intravenous methylprednisolone (30 mg/kg/day, maximum 1 g/day) was administered for five consecutive days after exclusion of CNS infection. Maintenance immunotherapy consisted of monthly IVIG and intravenous steroid pulses for 6-12 months, guided by clinical response. This regimen was chosen in accordance with prior reports in the literature [4,7].

Corticosteroid therapy was administered with careful monitoring for potential long-term adverse effects, along with pneumocystis jirovecii pneumonia (PJP) prophylaxis (TMP 5 mg/kg/day, maximum 160 mg/day), vitamin D and calcium supplementation, and a proton pump inhibitor. Routine immunizations were postponed during high-dose immunosuppressive therapy, in accordance with current pediatric immunization safety guidelines [4,8].

Follow-up and outcomes

By June 1, 2025, the OMS score had improved from 8 to 3, with complete resolution of opsoclonus and restoration of independent ambulation. In August 2025, the child was clinically stable, with normal gait, speech, and a full neurological examination while continuing monthly immunotherapy cycles. A follow-up FDG-PET scan is planned six months after the initial scan on May 28, 2025, to reassess for any occult or recurrent pathology, in accordance with previously published management protocols.

## Discussion

The child’s early anticholinergic triad, confusion, mydriasis with sluggish reactivity, and urinary retention, reasonably mimicked TCA ingestion; however, serious TCA poisoning typically presents with early cardiotoxicity (e.g., QRS prolongation) in addition to anticholinergic features, which were not evident here [9,10]. Concurrently, the marked CSF lymphocytic pleocytosis (235 cells/µL; ~90% lymphocytes) justifiably triggered a meningitis/encephalitis pathway, consistent with pediatric and Infectious Diseases Society of America (IDSA) guidance to treat empirically while diagnostic studies are pending and to consider autoimmune encephalitis when infectious studies are negative [11,12]. Notably, OMAS can show CSF immune activation (e.g., B-cell markers, oligoclonal bands) and occasional mild pleocytosis, reinforcing an immune-mediated process even when neuronal antibody panels are negative [13]. The decisive clinical turning point occurred during hospitalization when opsoclonus with evolving myoclonus and wide-based ataxia emerged. These features are not part of TCA toxidromes but are diagnostic hallmarks of pediatric OMAS. Contemporary reviews emphasize that OMAS can be diagnosed on clinical grounds when three of four core features are present (ocular opsoclonus; ataxia/myoclonus; sleep/behavioral disturbance; neuroblastoma) [14,15]. 

Pediatric OMAS is best understood as an immune-mediated, often paraneoplastic neuroinflammatory syndrome in which neuroblastoma acts as a trigger in 50% of cases, yet disease-defining serum/CSF neuronal antibodies are frequently absent. This profile fits our child’s negative panel [4]. Robust cellular immunology supports the diagnosis despite antibody negativity: CSF flow cytometry demonstrates marked B-cell enrichment and activated T-cell subsets that correlate with clinical severity [16]. Additional evidence of intrathecal immune activation includes CSF oligoclonal bands and neuroinflammation-linked biomarkers [17]. Cytokine network perturbations, with dysregulated B-cell activating factor (BAFF)/A proliferation-inducing ligand (APRIL) signaling and altered pro-/antiinflammatory cytokines, implicate both B-cell survival pathways and broader immune crosstalk [18]. Therapeutically, the biological relevance of B-cell pathology is underscored by rituximab studies showing sustained CSF B-cell depletion and clinical improvement, thereby linking mechanism to outcome [19]. Importantly, these immune signatures translate directly into clinical decision-making, supporting reliance on CSF cellular and intrathecal biomarkers when neuronal antibody panels are unrevealing. Large cohort data synthesize these findings, emphasizing OMAS as a neuroinflammatory disorder in which immune biomarkers (CSF B cells/oligoclonal bands (OCBs)) outperform commercial neuronal antibody panels for diagnostic support [1]. 

The relationship between OMAS and neuroblastoma is so robust that the presence of OMAS in a child is often considered a clinical mandate for a structured malignancy search. In our case, a comprehensive evaluation, including abdominal and pelvic ultrasound, FDG-PET/CT, and FDOPA-PET, revealed no evidence of adrenal or retroperitoneal mass and no abnormal metabolic uptake. Such negative findings are not uncommon, as neuroblastomas associated with OMAS may be small, mature, or even spontaneously regress, making them difficult to detect on initial imaging [13]. For this reason, expert consensus underscores the need for repeated surveillance, often at six to 12-month intervals, even when baseline scans are normal, to avoid missing an occult or late-presenting tumor [2]. 

First-line immunotherapy for pediatric OMAS combines high-dose corticosteroids (or adrenocorticotropic hormone (ACTH)) with IVIG; typical IVIG regimens are 2 g/kg over two to five days with consideration of repeated monthly dosing, and this approach improves neurological outcomes compared with steroids alone [3,4]. Because relapse risk and neurocognitive morbidity correlate with inadequately controlled inflammation, many centers employ maintenance immunotherapy, most often monthly IVIG and intermittent steroid pulses for 6-12 months, tailored to clinical response and tapered as stability is achieved [4,20]. Adjunct safety measures during prolonged/high-dose immunosuppression include Pneumocystis jirovecii pneumonia (PJP) prophylaxis, commonly with trimethoprim-sulfamethoxazole (TMP-SMX) in pediatric practice despite limited pediatric steroid-only guidance, and deferral of live vaccines, and in many policies, most immunizations during high-dose therapy, until glucocorticoids are tapered and B-cell-depleting effects have waned [8]. For refractory or relapsing disease, B-cell-directed therapy with rituximab has the strongest mechanistic and clinical signal in OMAS, producing rapid symptom reduction and sustained CSF B-cell depletion when added to steroids/IVIG or ACTH [1,19]. 

The imaging findings in our patient underscore the clinical value of repeat MRI in OMS. Although the first MRI (day 3) was unremarkable, the second MRI four weeks later revealed subtle T2/FLAIR/DIR hyperintense foci in peri-trigonal and subcortical white matter, as well as terminal zone changes consistent with delayed or evolving white matter signal abnormalities. These changes, in the absence of diffusion restriction, enhancement, or edema, were interpreted as nonspecific but may reflect underlying immunopathologic or demyelinating processes that evolve over time. In rare OMS and autoimmune encephalitis series, the timing of MRI can influence detection of such subtle lesions: one case notes that repeat imaging at one month is a practical interval to detect evolving or delayed MRI changes in neuroimmunologic syndromes [4,21,22].

This case illustrates the necessity of systematic reevaluation when clinical features evolve, underscoring the risk of anchoring bias in pediatric emergency, neurology, and toxicology settings. Awareness of OMAS is critical, as early recognition enables prompt immunotherapy, which improves prognosis. Malignancy screening must remain vigilant yet judicious, ensuring timely detection of neuroblastoma while minimizing unnecessary investigations. 

## Conclusions

This case highlights the diagnostic complexity of pediatric OMAS, which can initially mimic toxic or infectious etiologies before its distinctive features become evident. Continuous reassessment, structured malignancy screening, and timely initiation of immunotherapy are essential to optimize outcomes. Multidisciplinary awareness across emergency, neurology, and oncology settings is essential for early recognition, reducing misdiagnosis, and improving both neurological recovery and long-term prognosis.
